# Carbohydrates^☆^

**DOI:** 10.1016/j.advnut.2024.100311

**Published:** 2024-10-10

**Authors:** Joanne L Slavin, Sarah K Engstrom

**Affiliations:** 1 College of Food, Agricultural and Natural Resource Sciences, University of Minnesota, St. Paul, MN, United States; 2 Grande Custom Ingredients Group, Fond du Lac, WI, United States

## Nutrient

Carbohydrates include sugars and starches and are the predominant calorie source in most human diets, including grains, vegetables, fruits, dairy, legumes, and nuts. Additionally, dietary fiber, a class of nondigestible carbohydrates, is an essential nutrient for its role in chronic disease prevention [1,2]. Sugars are intrinsic in fruits and milk products. Sugars are also added to foods during processing and preparation or at the table. These “added sugars” sweeten the flavor of foods and beverages and improve their palatability [3]. Sugars are also used in food preservation and for functional properties such as viscosity, texture, body, and browning capacity [3]. In 2020, Food and Drug Administration (FDA) began enforcing Added Sugars labeling, distinguishing between Total and Added Sugars on Nutrition Facts panel.

Starches are made up of many glucose units linked together. They are found in many foods, including vegetables, legumes, and grains. Most starches are broken down to sugars by digestive enzymes in the body, but some starches are resistant to digestive enzymes. Fibers, such as starches, are made mostly of many sugar units bonded together. Unlike most starches, however, these bonds cannot be broken down by digestive enzymes and pass relatively intact into the large intestine. There, fiber can be fermented by the colonic microflora, or it can pass through the large intestine and bind water, increasing stool weight [4]. Although fibers are not converted to glucose, some short-chain fatty acids (SCFAs) are produced in the gut as fibers are fermented. SCFAs are absorbed and can be used for energy in the body. Fibers include both “dietary fiber,” the fiber naturally occurring in foods, and “functional fiber,” isolated fibers that have positive physiologic effects. Moving from a chemical to a physiological definition for dietary fiber creates challenges, with new FDA rules in 2016 requiring functional fibers to show a physiological benefit in human subjects before being accepted as a dietary fiber [5].

## Deficiencies

Calories can be obtained from both sugars and starches, so no deficiency of digestible carbohydrates is likely. Dietary fiber deficiency is common, with usual intakes of dietary fiber about half that of the recommended levels [1,6].

## Diet Recommendations

In its 2002 report [7], the Institute of Medicine (IOM) established a Recommended Dietary Allowance for carbohydrate of 130 g/d for adults and children aged ≥1 y. This value is based on the amount of sugars and starches required to provide the brain with an adequate supply of glucose. The IOM set an acceptable macronutrients distribution range for carbohydrates of 45%–65% of total calories. Thus, current dietary guidance recommends consumption of carbohydrate-containing foods, including grains, vegetables, fruits, pulses, nuts, seed, and milk products. Carbohydrate foods are an important source of dietary fiber and other nutrients.

Sugars and starches provide glucose, the main energy source for the brain, central nervous system, and red blood cells. Glucose can also be stored as glycogen (animal starch) in liver and muscle or, like all excess calories in the body, converted to body fat. Dietary fibers are nondigestible forms of carbohydrates. Dietary fiber is intrinsic and intact in plants, helps provide satiety, and promotes healthy laxation [4]. Diets high in fiber reduce the risk of coronary artery disease, diabetes, obesity, and other chronic diseases [1].

The energy value of digestible carbohydrates is generally accepted as 4 kcal/g for both sugars and starches. Fermentation of fiber in the gut will produce SCFAs that contribute calories, generally estimated to be ∼2 kcal/g.

In its 2002 report, the IOM set an adequate intake value for fiber of 14 g of fiber per 1000 kcal [7]. This value is derived from data on the relation of fiber consumption and coronary artery disease risk, although the IOM also considered the evidence for fiber decreasing the risk of chronic disease and other health-related conditions. Consequently, the IOM fiber recommendations are highest for populations who consume the most calories, namely young men. Fiber recommendations are lower for women and the elderly. The use of this method for determining recommend fiber intake for children is problematic (for example, intake of 19 g of fiber is recommended for 2-y-old children, an implausible number). More recent recommendations for children support practical recommendations as the “age plus 5” rule for fiber recommendations for small children (for example, 7 g fiber/d recommended for 2-y-old child) [8].

Dietary fiber is listed on the Nutrition Facts panel, and 28 g of dietary fiber is the currently recommended amount in a 2000-kcal diet. Manufacturers are allowed to call a food a “good source of fiber” if it contains 10% of the recommended amount of fiber and an “excellent source of fiber” if the food contains 20% of the recommended amount. Dietary fiber is required on food labels and includes both dietary fiber and functional fiber, whereas listing soluble and insoluble fiber is optional. Total carbohydrate, total sugars, and added sugars are also required on the Nutrition Facts panel.

## Food Sources

Grains, vegetables, fruits, legumes, milk, and milk products are the major food sources of carbohydrates. Grains and certain vegetables including corn and potatoes are rich in starch, whereas sweet potatoes are mostly sucrose, not starch. Fruits and dark-green vegetables contain little or no starch but provide sugars and some dietary fiber. Defining quality carbohydrate sources generally include plant food, whole-grain content, and dietary fiber, as well as providing nutrients of concern (potassium, vitamin D, calcium, and dietary fiber) [6]. Many high carbohydrate foods are also ultraprocessed foods, but still provide essential nutrients and meet standards for meeting the Dietary Guidelines for Americans [9]. Carbohydrate staple foods that are sustainable and important in cultural eating patterns should not be admonished for strict rules on added sugars or movement from enriched refined grains, which provide essential nutrients such as folic acid, other B vitamins, and iron to the diet [6].

## Clinical

Many clinical conditions require that individuals restrict carbohydrate intake to promote beneficial health outcomes. For example, lactose intake must be restricted for those with lactose intolerance, whereas diabetics must use carbohydrate exchange lists to monitor their digestible carbohydrate intake. Carbohydrates are often restricted in weight-loss diets—both as a means of cutting calories and to assist in more rapid weight loss. Conversely, athletes consume large quantities of digestible carbohydrate to fuel their activities. Thus, carbohydrate recommendation must be tailored for the person and lifestyle and to address individual nutrition needs.

## Toxicity

The Dietary Reference Intake (DRI) committee concluded that evidence was insufficient to set a Tolerable Upper Intake Level (UL) for carbohydrates [7]. However, a maximal intake level of ≤25% of total calories from added sugars was suggested by the panel. This suggestion was based on dietary intake survey data showing that children with diets at or above this level of added sugars were more likely to have poor intakes of essential nutrients including calcium. There is no UL for either dietary fiber or functional fiber.

Carbohydrates contribute to dental caries by providing substrate for bacterial fermentation in the mouth. Other justifications for limiting intake of added sugars include nutrient displacement and weight gain [10]. Recommendations of <10% of calories as added sugars are based on the Dietary Guidelines for Americans and are not included in the DRIs.

## Recent Research

As the DRIs recommend that 45%–65% of kcalories come from carbohydrates, it is challenging to find recent clinical studies that support lower intakes of digestible carbohydrates. Limits on added sugar should help reduce calorie intakes, but is there a physiological benefit for reducing added sugar if calories are not reduced?

Low carbohydrate diets, especially those that avoid grains, are a popular idea for weight control. Because carbohydrates provide more than half of the calories in a typical American diet, reducing carbohydrate intake will significantly decrease calorie intake, making it impossible to link improved health outcomes to low carbohydrates or low-calorie intake. There is significant clinical evidence supporting the beneficial effects of lower-carbohydrate dietary patterns on multiple established risk factors associated with insulin resistance and cardiovascular disease in adults [11]. Yet as the DRIs still recommend that carbohydrates comprise 45%–65% of calories in United States diets, it is difficult to get funding for feeding studies of low carbohydrate diets in healthy subjects, making data on this cohort limited.

Carbohydrates are less expensive than proteins and fats as a source of calories, and carbohydrate staple foods are important cultural foods. Societies around the globe have incorporated carbohydrate staples as the foundations of their diets. Potatoes in Ireland, pasta in Italy, tortillas in Mexico, rice in Asia, or bread in France are all examples of how we build diets around carbohydrate staples at the base of our diet pyramid.

All portions of myplate.gov include carbohydrates especially dietary fiber. Foods that bring along our nutrients of concern, dietary fiber, vitamin D, calcium, and potassium, are also recommended in dietary guidance. Additionally, enriched refined grains, considered ultraprocessed, are important contributors of folic acid, B vitamins, and iron in United States diets. Carbohydrate intake must be tailored to the individual and be respectful to cultural eating practices and food costs.

## Author contributions

The authors’ responsibilities were as follows – JLS, SKE: were responsible for design, writing, and final content of the article and JLS, SKE: read and approved the manuscript.

## Funding

The authors reported no funding received for this study.

## Conflict of interest

The authors report no conflicts of interest.
